# Self-Reported Knee Symptoms Assessed by KOOS Questionnaire in Downhill Runners (Skyrunners)

**DOI:** 10.1371/journal.pone.0126382

**Published:** 2015-04-22

**Authors:** Giulio Sergio Roi, Marco Monticone, Marco Salvoni, Roberto Sassi, Giampietro Alberti

**Affiliations:** 1 Isokinetic Medical Group, Education and Research Department, Bologna, Italy; 2 International Skyrunning Federation, Biella, Italy; 3 Physical Medicine and Rehabilitation Unit, Scientific Institute of Lissone (Milan), Institute of Care and Research (IRCCS), Salvatore Maugeri Foundation IRCCS, Lissone, Italy; 4 Department of Computer Science, University of Milan, Milan, Italy; 5 Department of Biomedical Sciences for Health, University of Milan, Milan, Italy; Queen Mary University of London, UNITED KINGDOM

## Abstract

**Background:**

The knee is the weight-bearing joint most commonly associated with sports injuries, and therefore is most at risk of developing degenerative changes, including osteoarthritis. Skyrunners can be considered to be at risk of developing symptoms of post-traumatic osteoarthritis due to downhill running.

**Aim:**

The aim of this study was to analyze the health of the knee joints of a large group of these athletes via a specific self-report questionnaire.

**Methods:**

This study was carried out by asking the participants of seven official Skyraces (22.4±3.1 km length; 1596±393 m elevation) to fill out a questionnaire. Information regarding age, sex, downhill elevation (m) during training and competitions over the last month, and history of previous knee injury was also collected before the participants filled out the Knee injury and Osteoarthritis Outcome Score (KOOS), which is a reliable and validated instrument designed to assess patients’ opinions about their knees and associated problems that can result in post-traumatic osteoarthritis. Athletes were divided into six age groups (from 17 to 70 years) and 12 groups based on the downhill gradient they had covered over the last month (from 1,000 to 40,000 m).

**Results:**

Six hundred twenty-one questionnaires were collected from 45% of the participants in the seven races. Multivariate analysis revealed that self-reported KOOS scores were unrelated to age, sex and monthly downhill gradient. Only 74 (12%) of the participants reported previous knee injuries. Significant differences in the five subscales of the KOOS were found between skyrunners with and without previous knee injuries (P<0.01).

**Conclusions:**

In the studied population, regular training for downhill running and participation in Skyraces could not be considered risk factors for subjective knee symptoms. Skyrunners with self-reported histories of knee injuries scored worse on all five subscales of the KOOS.

## Introduction

Skyrunning is a sporting activity performed in the mountains at altitudes of 2,000 m or more above sea level on uphill and downhill tracks with gradients of 30–40% [1]. The most popular skyrunning competitions are called Skyraces, which are defined as competitions at altitude that cover distances of 20 to 30 km (International Skyrunning Federation, Rule 2.4.3) (Table 1). In these competitions, the vertical uphill speeds attained by the athletes are always lower than the downhill speeds, and these speeds are the result of the individual characteristics of the athletes who must be supported by adequate training that is performed both on uphill and downhill tracks. For example, in the Dolomites Skyrace, a classic competition with consecutive uphill and downhill tracks that cover over 1,750 m of elevation (uphill: 10 km, 18% mean incline; downhill 12 km, 14% mean decline), the overall best performances were 1 h 16 min 47 s and 43 min 35 s for the uphill and downhill tracks, respectively. Thus, the average horizontal speeds were 7.8 and 16.5 km/h, and the average vertical speeds were 1,367 and 2,409 m elevation per hour for the uphill and downhill tracks, respectively (for females these records were 14 and 26% slower).

**Table 1:** Characteristics of the Skyraces.

Race	Total distance	Total downhill elevation	Fastest time	Slowest time	Participants	New Participants	Questionnaires	
	km	m	h:mm:ss	h:mm:ss	N°	N°	N°	Partial Sum
**Valetudo Skyrunning**	23.1	1350	1:49:58	4:00:04	230	230	97	97
**SkyRace Quattro Passi**	23.0	1450	2:07:17	4:28:49	163	148	75	172
**SkyRace Casere**	24.0	1850	2:17:25	4:33:13	98	69	61	233
**International SkyRace Carnia**	24.3	2004	2:34:33	5:48:46	178	171	92	325
**Stava SkyRace**	21.5	2100	2:07:49	4:36:45	171	121	87	412
**Dolomites SkyRace** *	16.3	1385	1:35:34	3:32:59	618	508	135	547
**SkyRace Ortles—Cevedale**	25.9	1035	2:13.59	5:28:30	193	119	74	621
**Mean±SD**	22.4±3.1	1596±393	2:06:39±0:19:07	4:38:27±0:47:08	236±173	195±147	89±24	**—**

Characteristics of the Skyraces, times taken to cover the total race distance by the winners and the last racers, number of questionnaires administered in each competition and number of participants. New participants are calculated in respect to the previous races.

*Race shortened for security because of bad weather.

While uphill running requires energy expenditure to elevate the body’s center of mass, downhill running utilizes the potential energy accumulated during the uphill run and thus requires less energy expenditure (e.g. in the slope range of 20–40% the vertical cost of running attains a minimum value of 44.9±3.8 J/kg/m in uphill and of 9.2±1.7 J/kg/m in downhill [1]), and that energy is utilized to decelerate and control body position and also to maintain or increase speed if possible. During such locomotion activity, muscle contractions help to protect joints from impact loads [2], and the role of the eccentric muscular actions of the lower limbs is particularly important for absorbing energy [3] and stabilizing the joints [4].

It is well known that increased joint use and impact loading can cause articular surface injuries [3]; moreover, competitive athletes performing high impact sports sustain a wide variety of soft tissue, bone, ligament, tendon, and nerve injuries, caused by direct trauma or repetitive stress. The knee is the weight-bearing joint most commonly associated with sports injuries, and therefore is most at risk of developing degenerative changes, including osteoarthritis (OA), more common among former athletes, compared with the general population [5]. However, only a few studies provide insight into the relationship between regular vigorous sporting activities such as skyrunning and the development of symptoms of joint degeneration [6, 7, 8].

Due to the speeds attained in downhill running, participants in Skyraces can be considered at risk for the development of symptoms of post-traumatic osteoarthritis. The aim of this study was to analyze the health of the knee joints of a large group of these athletes via a specific self-report questionnaire.

## Methods

### Subjects

The subjects of this study were recruited from the 1,651 participants in the 2010 season of seven official Skyraces at the national and international levels (Table 1). Recruitment was performed the day before the races during the briefing meetings or after the end of the races during the lunches or the prize ceremonies. The only one criteria for inclusion/exclusion was the participation in the races, checked from the official starting lists delivered by the organizers. These lists included only male and female of 18 years of age and over, who must submit a valid medical certificate of fitness for skyrunning competitions before registration.

### Ethics Statement

The study was approved by the authors' Institutional Review Board (Scientific Committee of the International Skyrunning Federation), and has been conducted according to the principles expressed in the Declaration of Helsinki.

### Questionnaire

Subjects were asked to fill out the Knee injury and Osteoarthritis Outcome Score (KOOS). If the subjects agreed to participate, each subject provided written informed consent prior to filling out the questionnaire. Each athlete responded to the questionnaire only once. Upon delivery of the questionnaire, an investigator ensured that all items were completed, and in cases of missing items, the investigator asked the subject to complete all items of the questionnaire; thus, all questionnaires were completely filled out.

Before filling the questionnaire, athletes were asked to give information regarding age, sex, downhill elevation (m) covered in training and in competitions over the last month, and history of previous knee injuries.

The KOOS was developed as a reliable and valid instrument to assess patients’ opinions about their knees and associated problems that can result in post-traumatic osteoarthritis [9, 10]. It has been used in men and women ranging from 14 to 79 years in age with varying disorders resulting in knee complaints such as mild, moderate and severe OA, anterior cruciate ligament tear and meniscus tear [9, 10, 11].

The KOOS is a self-administered 42-item questionnaire that requires approximately ten minutes to complete. It consists of the following five subscales: pain, other symptoms, function in daily living (ADL), function in sports and recreation (Sport/Rec) and knee-related quality of life (QOL). The previous week of activity/training is taken into consideration when answering the questions. Standardized answer options are given (i.e., five Likert boxes), and each question is scored from 0 to 4. A normalized score (100 indicates no symptoms, and 0 indicates extreme symptoms) is then calculated for each subscale. We used the Italian version of the KOOS, which previous research indicated to be reliable and valid in subjects with knee injuries [12].

### Statistics

Statistical analyses were performed with the IBM SPSS Statistics (IBM, New York, NY, USA) version 20 software package. Athletes were arbitrarily divided into six age independent groups (17–20; 21–30; 31–40; 41–50; 51–60; 61–70 years) and into 12 independent groups based on the downhill gradient they had covered in the last month of activity/training (0–999; 1,000–1,999; 2,000–2,999; 3,000–3,999; 4,000–4,999; 5,000–5,999; 6,000–6,999; 7,000–7,999; 8,000–9,999; 10,000–14,999; 15,000–19,999; 20,000–39,999 m) (between-subjects design). The group partition levels and the number of subjects included in each class are reported in the first two rows of Tables 2 and 3.

**Table 2:** KOOS scales by age group.

Age group (yrs)	17–20	21–30	31–40	41–50	51–60	61–70	H, χ^2^(5)	*p*
**N°**	12	108	253	184	61	3	—	—
**Mean age (yrs)**	19 ± 1 (18–19)	27 ± 3 (26–27)	36 ± 3 (35–36)	45 ± 3 (45–46)	54 ± 3 (54–55)	64 ± 4 (56–73)	—	—
**Pain**	90 ± 9 (84–96)	90 ± 12 (88–92)	90 ± 12 (88–91)	89 ± 14 (87–91)	90 ± 12 (87–93)	83 ± 19 (36–130)	1.236	0.941
**Symptoms**	91 ± 10 (85–97)	88 ± 12 (86–91)	91 ± 10 (90–92)	91 ± 10 (90–93)	92 ± 10 (89–95)	88 ± 6 (74–102)	9.000	0.109
**ADL**	95 ± 7 (90–99)	97 ± 6 (95–98)	95 ± 9 (94–97)	95 ± 10 (94–96)	95 ± 9 (93–98)	91 ± 14 57–125)	2.123	0.832
**Sport/Rec**	83 ± 17 (72–94)	85 ± 16 (83–88)	88 ± 15 (87–90)	87 ± 16 (85–89)	87 ± 17 (83–92)	75 ± 31 (0–153)	5.489	0.359
**QOL**	85 ± 10 (79–92)	84 ± 17 (80–87)	83 ± 18 (81–85)	84 ± 16 (82–86)	84 ± 17 (80–88)	83 ± 24 (24–143)	0.418	0.995

Mean ± SD scores in the five scales and 95% CI by age groups. H is the Kruskal-Wallis statistic, approximatively distributed as **χ** ^**2**^ (5). Bonferroni corrections for multiple comparisons were employed (statistical significant differences are found for p<0.01).

**Table 3:** KOOS scales by monthly downhill gradient.

Gradient (m)	0–999 m	1,000–1,999 m	2,000–2,999 m	3,000–3,999 m	4,000–4,999 m	5,000–5,999 m	6,000–6,999 m	7,000–7,999 m	8,000–9,999 m	10,000–14,999 m	15,000–19,999 m	20,000–39,999 m	H, χ^2^(11)	*p*
**N°**	66	81	83	92	59	66	27	21	35	62	13	16		
**Pain**	90±11 (87–93)	89±15 (86–93)	90±11 (88–93)	89±15 (85–92)	91±10 (88–94)	89±13 (86–92)	88±11 (84–93)	86±14 (80–93)	91±12 (87–95)	89±14 (86–93)	90±13 (82–97)	90±8 (86–95)	4.562	0.65972
**Symptoms**	91±12 (88–94)	92±10 (89–94)	91±11 (88–93)	90±10 (88–92)	91±9 (89–93)	91±9 (89–93)	91±9 (87–94)	90±11 (85–96)	92±9 (89–95)	89±13 (86–92)	93±9 (88–98)	91±11 (85–97)	4.999	0.64653
**ADL**	96±6 (95–98)	95±10 (93–98)	95±10 (84–91)	94±11 (92–97)	96 ±6 (94–98)	96±7 (94–97)	97±4 (96–99)	94±12 (88–99)	98±5 (96–99)	95±8 (93–97)	98±2 (97–100)	96±8 (91–100)	5.725	0.61875
**Sport/Rec**	86±15 (82–90)	89±17 (85–92)	88±16 (84–91)	86±18 (82–89)	88 ±11 (85–91)	86±14 (82–89)	89±11 (85–94)	84±20 (75–93)	91±14 (86–96)	86±19 (81–91)	87±12 (80–94)	92±11 (86–97)	12.849	0.21042
**QOL**	82±19 (78–87)	84±20 (80–87)	84±16 (81–88)	82±17 (78–85)	84 ±16 (80–88)	84±16 (80–88)	85±13 (80–90)	82±22 (73–92)	85±15 (80–90)	84±16 (80–88)	86±14 (78–95)	82±17 (73–91)	3.759	0.67778

Mean ± SD scores in the five scales and 95% CI, by monthly downhill gradient. H is the Kruskal-Wallis statistic, approximatively distributed as χ2 (11). Bonferroni corrections for multiple comparisons were employed (statistical significant differences are found for p<0.01).

Univariate comparisons of each of the five KOOS scores between groups were performed with the Kruskal-Wallis H test, given the fact that the assumption of normality was not satisfied for every group combinations of KOOS scores, as assessed by Shapiro-Wilk's test (p≤0.05). Distributions of KOOS scores were similar for all groups, as assessed by visual inspection of a boxplot. Bonferroni corrections for multiple comparisons were used, thus values of p<0.05 / 5 = 0.01 were deemed significant.

Multiple regression was employed to study the variation of each of the KOOS scores as explained by the four ordinal factors considered (age group, sex monthly gradient group and self-reported previous history of knee injury), as well as the contribution of each predictor to the total. Independence of residuals was assessed by Durbin-Watson statistics. A value of p<0.05 was considered significant.

## Results

One thousand three hundred sixty-six different skyrunners participated in the seven races, and 285 of these skyrunners participated in more than one race. Forty-one runners did not finish their race (3%), and only two reported injuries during downhill running (one ankle sprain and one deep wound in the thigh caused by a fall).

We collected 621 questionnaires, which corresponded to 45% of the participants (Table 1).

### Age-related differences

The mean age of the skyrunners who filled out the questionnaire was 38.6±9.2 years (range 17–68). There were no significant differences (p>0.01) between the six age groups in the five subscales of the KOOS (Table 2).

### Gradient-related differences

There were no significant differences (p>0.01) between the twelve gradient groups in the five subscales of the KOOS (Table 3).

### Sex differences

There were no significant differences (p>0.01) between the sexes in the five subscales of the KOOS (Table 4).

**Table 4:** KOOS scales by sex.

Previous knee injuries	Females	Males	H, χ^2^(1)	*p*
**N°**	85	536	—	—
**Pain**	91 ± 12 (89–94)	89 ± 13 (88–90)	2.108	0.147
**Symptoms**	91 ± 13 (88–94)	91 ± 10 (90–92)	1.368	0.242
**ADL**	96 ± 9 (94–98)	95 ± 8 (95–96)	0.807	0.369
**Sport/Rec**	89 ± 16 (86–92)	87 ± 16 (86–88)	2.209	0.135
**QOL**	84 ± 19 (80–88)	84 ± 17 (82–85)	0.793	0.373

Mean ± SD scores in the five scales and 95% CI of female and male skyrunners. H is the Kruskal-Wallis statistic, approximatively distributed as χ2 (1). Bonferroni corrections for multiple comparisons were employed (statistical significant differences are found for p<0.01).

### Differences due to previous knee injuries

Only 74 (12%) of the participants reported previous knee injuries: 27 of them (4%) reported that they were treated by surgery (13 anterior cruciate ligament reconstructions, seven meniscal tears, three cartilage, four other), and the other 47 (8%) reported that their injuries were treated conservatively (18 tendinopaties, nine cartilage injuries, eigth contusions, five meniscal pathologies, seven other). Significant differences (p<0.001) were found between skyrunners with and without previous knee injuries in the five subscales of the KOOS (Table 5).

**Table 5:** KOOS scales by previous knee injuries.

Previous knee injuries	YES	NO	H, χ^2^(1)	*p*
**N°**	74	547	—	—
**Pain**	80 ± 17 (76–84)	91 ± 12 (90–92)	36.467	<0.0001
**Symptoms**	85 ± 14 (82–88)	92 ± 10 (91–92)	24.867	<0.0001
**ADL**	92 ± 11 (89–94)	96 ± 8 (95–97)	25.621	<0.0001
**Sport/Rec**	76 ± 19 (72–81)	89 ± 15 (87–90)	38.011	<0.0001
**QOL**	68 ± 20 (63–72)	86 ± 15 (85–87)	58.909	<0.0001

Mean ± SD scores in the five scales and 95% CI in participants with (YES) and without (NO) self reported knee injuries. H is the Kruskal-Wallis statistic, approximatively distributed as χ^2^ (1). Bonferroni corrections for multiple comparisons were employed (statistical significant differences are found for p<0.01).

### Multivariate analysis

In a multiple regression analysis that included as predictors for each of the five KOOS subscales: age group, sex, downhill gradient (run over the last month) group, and self-reported history of previous knee injuries, only the latter was associated with significantly lower scores in all five KOOS subscales (p<0.001). However, previous knee injuries explained, at most, 11.5% of the variance in any of the five KOOS subscales (Table 6).

**Table 6:** Factors influencing KOOS scales.

	Sex	History of knee injury	Age group	Monthly gradient group	adj. *R* ^*2*^	Durbin-Watson
**Pain**	1.714 (1.455)	-10.429*** (1.537)	-0.415 (0.529)	-0.012 (0.162)	0.067	1.830
**Symptoms**	0.216 (1.190)	-6.643*** (1.257)	0.757 (0.432)	-0.008 (0.132)	0.042	2.045
**ADL**	0.228 (0.996)	-4.198*** (1.052)	-0.381 (0.362)	0.090 (0.111)	0.022	1.929
**Sport/Rec**	1.906 (1.791)	-12.324*** (1.892)	0.401 (0.651)	0.155 (0.199)	0.061	1.976
**QOL**	0.036 (1.872)	-18.135*** (1.977)	0.157 (0.680)	0.0151 (0.208)	0.115	2.036

Summary of multiple regression analysis showing potential factors influencing self reported KOOS values (N = 621). Rows contain separate analyses. Unstandardized regression coefficients and their Standard Errors (within brackets) are reported.

***p≤0.001. adj. R^2^ is the adjusted coefficient of determination. The Durbin-Watson statistics is also reported (independence of residuals).

## Discussion

The aim of this study was to analyze the health of the knee joints of athletes involved in skyrunning competitions via the KOOS questionnaire. The main results indicate that in these athletes KOOS scores were unrelated to age, sex and monthly downhill gradient, and that Skyrunners with self-reported histories of knee injuries scored worse on all five subscales of the questionnaire.

In the literature, there are some reports on the dangers of articular surface injury that accompany increased joint use and impact loading [4]. Compared to level running, at 15% decline the normal impact peak and the parallel braking force peaks increase by 54% and 73% respectively [13]. Specifically, intense physical activity and higher impact forces during downhill running have been observed to lead to acute and/or chronic injuries [13] that result in decreased knee function. However, in this cross-sectional study of a large sample of skyrunners, scores on the five subscales of the KOOS were found to be unrelated to the monthly downhill gradients covered by the skyrunners (Tables 3 and 6), indicating that regular training for downhill running *per se* cannot be considered a risk factor for subjective symptoms of chronic injuries in this population of participants to competitions. This result is in accordance with previous studies in which the hypothesis that long distance running is associated with increased incidences and severities of OA was rejected [6, 7, 8, 14, 15]. Consequently, the beliefs that the major cause of OA is “wear and tear” and that OA is caused and exacerbated by exercise should be rejected [14] and also should not be considered valid for downhill runners. Currently, OA is thought to be a complex syndrome with multiple causes and a constellation of symptoms and signs [16] in which muscle dysfunction [14, 17] and individual injury threshold limits [18, 19] most likely play relevant roles. This result is further confirmed based on the considerations that, during downhill running, muscle forces are larger and involve greater co-activation, which increases joint stiffness. Co-activation reduces the net work required by each joint [20], and impact forces can be lowered by increasing knee flexion angles at foot strike and decreasing stride lengths [21]. Furthermore, there are other safety mechanisms of neuromuscular origin that are involved in downhill running such as muscle pre-activation [22] and the stretch reflex [23].

In downhill running, it is known that the peak flexion angle of the knee is greater at footstrike compared to level running [24]. Thus, the eccentric work is performed at a greater muscular length, which produces greater muscle damage of mechanical origin [25] and leads to delayed-onset muscle soreness and impairment of strength-generating capacity [25]. However, specific training (i.e., downhill running) may attenuate soreness, tenderness and the clinical signs of fatigue, and muscle damage [25]. Pierrynowski et al. [26] found that as little as two 12-minute bouts of downhill running at a -10% gradient are sufficient to protect against the occurrence of delayed-onset muscle soreness after a subsequent downhill run three days later.

It is also well known that the metabolic energy cost of downhill running is less than that of level or uphill running at the same speed [1] and that athletes specifically trained in mountain running develop a more economical style than nonathletic subjects. This allows for better recovery of the elastic energy for braking produced by eccentric muscular work during descent [1, 3]. However, the running speeds adopted in downhill competitions are far lower than metabolically feasible speeds, and on extreme downhill gradients, skyrunners always choose to run slower than metabolically possible mainly because of safety concerns that operate to minimize joint and tissue injuries [1].

This study also indicated that self-reported KOOS scores were unrelated to the age and sex of the skyrunners (Table 6). This result is in accordance with those of Frobell et al. [27] who reported that age, sex, and body mass index were not related to KOOS subscale scores in 188 amateur football players ranging from 14 to 39 years of age.

Previous studies have found that knee symptoms and functional difficulties increase with age in healthy sedentary populations [28, 29], and this decline in function is most apparent in the Sport and Recreation subscale [29]. We did not find the same trend in skyrunners; indeed, all subscale scores were stable across age groups (Table 3). This finding is likely related to the fact that skyrunners of all ages are more physically active than matched sedentary people.

Taken together, these observations lead to the conclusion that downhill running may have a protective effect on the joints due to its eccentric component. This protective effect is supported by the widespread utilization of eccentric exercises in orthopedics and sports rehabilitation [2] in which moderate inclined slopes appear to be a safe component of training regimens and post-injury protocols aimed at returning the participant to running [30]. A protective effect was found also in a recent study of rats demonstrating that the joint loading induced by eccentric muscular actions during downhill running leads to site-specific adaptations in cartilage tissue [31]. A 10-week study employing gadolinium-enhanced magnetic resonance imaging of cartilage (dGEMRIC) found condroprotective effects on the knee that are useful for OA prevention in female novice runners who jogged up to 5 km [32]. Despite these findings, the above-mentioned conclusion needs to be thoroughly confirmed by well-designed longitudinal studies of athletes and mountain runners.

In this study, 12% of the skyrunners reported histories of knee injury. These skyrunners scored worse on all five subscales of the KOOS (Table 5). The median differences between skyrunners with and without previous knee injuries were clinically significant because skyrunners with previous knee injuries scored more than 8–10 points [33] lower on the pain, sport/rec, and knee-related quality of life subscales. Thus, we conclude that the association between a previous history of knee injury and lower KOOS scores may constitute a risk factor for the development of subjective symptoms of OA.

Similar findings have been described for amateur football players [27]; those with self-reported previous knee injuries score worse on all five subscales. This is most likely a consequence of the higher incidence of traumatic knee injuries in football, which is classified as a contact sport [34].

In 1990, Konradsen et al. [7] studied 30 Danish male orienteering runners who had qualified for county teams between 1950 and 1955. These authors reported that 22% of the 27 who remained active after approximately 40 years of practice experienced intermittent pain in the joints of their lower extremities while running, and only one of the three former runners who was no longer active stopped running because of multi-joint OA with grade 3 OA in the knees. These authors concluded that 40 years of running an average of 20–40 km/week does not lead to OA degeneration in individuals without underlying problems resulting from pre-existing lower extremity injuries.

Shirer [14] proposed three possible mechanisms of cartilage damage by which previous injury could increase the risk of OA: i) cartilage damage occurs at the time of the injury, and OA develops over subsequent years; ii) cartilage damage is a consequence of joint instability after ligament injury; and iii) the muscle dysfunction associated with injury leads to recurrent cartilage damage because impact forces are no longer being absorbed appropriately. This author concluded that a wide variety of sports, not including recreational sports, are associated with OA.

The association between sport and OA can be especially strong in team sports and sporting activities in which technical and tactical skills are the main determinants of performance, so athletes often can play while injured. In contrast, sports where performance is mainly determined by endurance training, the participation while injured is usually avoided because injury can strongly affects both training and performance. These observations need further studies, however the injury-associated muscle dysfunction hypothesis indicates that proper rehabilitation after injury may be important in the prevention of OA [14].

It is interesting to note that, despite previous knee injuries and lower KOOS scores, almost all skyrunners were able finish all of the studied Skyraces, and the occurrence of athletes being forced to withdraw from these competitions because of injury is very rare [35, 36]. This means that these athletes are relatively symptom free and maintain a high level of activity and performance, but we cannot exclude OA or chronic knee injury only from the absence of subjective symptoms.

There are some limits that affect the power of this study. First, there was a selection bias because we collected questionnaires only from subjects participating in the races; thus, all skyrunners who were unable to participate, including those affected by knee problems, were not considered. Second, a recall bias was introduced by the self-reporting of knee injury histories. Furthermore, we only administered the KOOS questionnaire and did not collect other diagnostic data (e.g., imaging); thus, we were unable to correlate KOOS scores with specific diseases or injuries, particularly OA, though KOOS was demonstrated to be reliable and valid instrument to assess patients’ opinions about their knees and associated problems that can result in post-traumatic osteoarthritis [9, 10]. Finally, this study employed a cross-sectional design and therefore provides information about the prevalence of self-reported knee problems in skyrunners, but the true incidence of knee problems and the relationships of those problems with other variables should be more rigorously analyzed in longitudinal studies that would entail imaging and/or physical exam findings and possibly capturing the whole gamut of participants.

## Conclusions

Self-reported KOOS scores from a large sample of skyrunners were found to be unrelated to age, sex, and monthly downhill gradient covered. These findings indicate that regular training for downhill running and participation in Skyraces cannot be considered to be risk factors for subjective symptoms of chronic injuries and OA.

During downhill running, athletes adopt protective mechanisms of neuromuscular origin, including muscle co-activation, pre-activation, and the stretch reflex, that increase joint stiffness and reduce the net work required of each joint. Furthermore, the increase in knee flexion angle at footstrike and the decrease of stride length lower the impact forces. Eccentric muscular work plays a crucial role in these mechanisms because it seems to be involved in site-specific cartilage adaptation.

Skyrunners with self-reported histories of knee injuries scored worse on all five subscales of the KOOS, suggesting that a history of knee injury most likely constitutes a risk factor for subjective symptoms of OA; thus, proper rehabilitation after injury may be important for the prevention of OA.

Longitudinal studies with much more objective measures that would entail imaging and/or physical exam findings, are necessary to confirm the results and the conclusions of this study, and to better understand the limits within which downhill running can safely be performed.
